# Intervention for promoting intake of fruits and vegetables in Brazilians: a randomised controlled trial

**DOI:** 10.1017/S1368980021004341

**Published:** 2022-03

**Authors:** Raquel de Deus Mendonça, Sueli Aparecida Mingoti, Maria Flávia Gazzinelli Bethony, Miguel Angel Martinez-Gonzalez, Maira Bes-Rastrollo, Aline Cristine Souza Lopes

**Affiliations:** 1 Department of Nutrition, Universidade Federal de Minas Gerais, Avenue Alfredo Balena, 190, Belo Horizonte, MG 30190-100, Brazil; 2 CAPES Coordination for the Improvement of Higher Education Personnel Foundation, Ministry of Education of Brazil, Setor Bancário Norte (SBN), Quadra 2, Edifício CAPES, Brasília, DF, Brazil; 3 Department of Statistics, Universidade Federal de Minas Gerais, Belo Horizonte, MG, Brazil; 4 Department of Applied Nursing, Universidade Federal de Minas Gerais, Belo Horizonte, MG, Brazil; 5 Department of Preventive Medicine and Public Health, University of Navarra, Pamplona, Navarra, Spain; 6 Department of Nutrition, Harvard TH Chan School of Public Health, Boston, MA, USA

**Keywords:** Fruit, Vegetables, Feeding behaviour, Nutrition education, Brazil

## Abstract

**Objective:**

To evaluate the effectiveness of a nutritional intervention to promote fruit and vegetable (FV) intake.

**Design:**

A randomised controlled community trial was conducted to evaluate the effectiveness of a 7-month nutritional intervention and to promote FV intake, separately and together. All participants attended physical exercise sessions. The intervention was based on the transtheoretical model and Paulo Freire’s pedagogy. The interventions included group educational sessions, motivational cards and informational materials. The primary outcome was a change in FV intake (g/d), and secondary outcomes included stages of change, self-efficacy, decisional balance and knowledge on FV. All data were collected face-to-face; and FV intake was assessed using a validated brief questionnaire.

**Setting:**

Health promotion services of Brazilian Primary Health Care.

**Participants:**

3414 users of Brazilian Primary Health Care (1931 in the control group and 1483 in the intervention group (IG)).

**Results:**

At baseline, the average daily FV intake was 370·4 g/d (95 % CI 364·2, 376·6). The increase in FV intake (23·4 g/d; 95 % CI 6·7, 40·0) and fruit intake (+17·3 g/d; 95 % CI 5·1, 29·4; *P* = 0·01) was greater in the IG among participants in the lowest baseline intake. Participants in the IG also showed progression in the stages of change (*P* < 0·001), increased self-efficacy (*P* < 0·001) and improved knowledge of FV crops (*P* < 0·001).

**Conclusions:**

The nutritional intervention was effective in increasing FV intake and fruits intake among individuals with a lower intake at baseline and in maintaining FV intake among those who reported consuming FV as recommended (400 g/d).

Suboptimal fruit and vegetable (FV) intake is one of the 10 main contributors to the global burden of disease^(1)^. FV intake is associated with a lower risk of mortality and helps to prevent conditions such as obesity, diabetes, CVD, cancer and mortality^(1–3)^. However, an inadequate FV intake is notable globally, and the intake of FV is generally low in relation to the recommended amount (>400 g or 5 servings per day)^(1)^.

According to the European Union, approximately 12 % of adults consume 5 servings (≅400 g) of FV daily, and people with higher education levels tend to eat more FV than those with lower education levels^(4)^. In the USA, only 12·2 % and 9·3 % of adults consume FVs, respectively, in accordance with the recommended amounts^(5)^. In Brazil, a National Health Survey showed that only 37·3 % of adults consumed 5 servings of FV per day^(6)^. One study, that assessed the food patterns in 18 countries, showed that the participants consumed an average of 3·8 servings (≅304 g) of FV per day; and FV intake in low-income and lower-middle-income countries were 2·1 and 3·2 servings per day (≅168 g and 256 g of FV), respectively^(7)^.

WHO suggests performing effective, low-cost interventions that aim to increase FV intake^(1)^. Promising results have been documented in a number of studies^(8–10)^. However, most trials have been conducted in developed countries, and intervention studies have rarely been conducted in poor communities. A systematic review of interventions and programmes, promoting fruit and/or vegetable intake in adults, showed that only 5 out of 44 studies included low-income participants^(11)^. Similarly, in another systematic review of interventions, based on feeding behaviour theories, 7 out of 34 studies were conducted among minority and low-income populations^(12)^. Systematic reviews have also shown that few papers reported the theories and methods used in the interventions^(11–13)^. Additionally, most of the nutritional interventions did not examine the individual’s perception of intake^(14)^.

Furthermore, few studies have been conducted in the health services^(15)^, which might affect the sustainability of outcomes. The promotion of adequate and healthy food, including the intake of FV, is the focus of several Brazilian public policies. This includes the implementation of the Food Guide, which is one of the priorities of health services, mainly in Primary Health Care. However, it is still necessary to direct local strategies for promoting the intake of FV, and respecting the food culture of each region in Brazil^(16)^.

Therefore, this study was designed to promote FV intake and was based on the perceptions of low-income participants regarding their intake habits and using theories. Thus, the study aimed to evaluate the effectiveness of nutritional interventions in promoting FV intake among participants of the health promotion services from the Brazilian Primary Health Care.

## Methods

### Study design and setting

This study was a community randomised controlled trial, conducted to promote FV intake in a representative sample of participants from the Health Academy Programme (HAP) in Belo Horizonte, Minas Gerais, Brazil, between February 2013 and March 2015.

Belo Horizonte is the capital of the Minas Gerais state, located in Southeast Brazil. It is the sixth most populous city in the country, with an estimated population of 2·5 million inhabitants and a high Human Development Index value of 0·810^(17)^. For health action planning, the city is divided into 9 administrative regions.

This municipality has focused on strategies to enhance public health policies and inter-sector actions for health promotion in more socially vulnerable populations, with a heavy investment in primary care. For this, it created the HAP, in 2006, as a health programme integrated with the Primary Health Care network of the national healthcare system^(18)^. HAP consists of a set of facilities with infrastructure, equipment and qualified professionals to guide the physical activity and the promotion of healthy dietary habits, at no cost to participants.

### Study population

From the 50 units of HAP operating in Belo Horizonte at the time of sampling, 42 centres were at eligible locations with regard to community health vulnerability. The eligibility criteria for centres were: located in areas with high Health Vulnerability Indexes (priority given for the implementation of HAP by the municipality), had morning operation hours and had not participated in nutrition intervention activities in the past 2 years. The Health Vulnerability Index is a composite index that incorporates aspects of sanitation, housing, education, income and health. The Health Vulnerability Index is a combination of different variables within an indicator that may be used to summarise relevant information that reflects intra-urban inequalities. The indicators represent basic sanitation, housing, income and health, and are scored from 0 to 1, and the closer the value is to 1 (1·0), the greater the health vulnerability of the population; very low (0 to 0·200), low (0·201 to 0·300), medium (0·301 to 0·400), high (0·401 to 0·500) or very high (0·501 to 1)^(19,20)^.

A total of 18 centres were randomly selected and consisted of 2 centres per administrative district. The centres were randomly divided into the intervention group (IG) or control group (CG), randomised to provide a power of 80 % to detect a change in FV intake in the IG. All participants were not blinded to the group allocations. These centres were representative of the HAP programme with a 95 % CI and < 1·4 % error based on an estimation of the HAP population proportion (sex, age, education level). Details of the sampling process are available in a previous paper^(19)^.

**Fig. 1:**
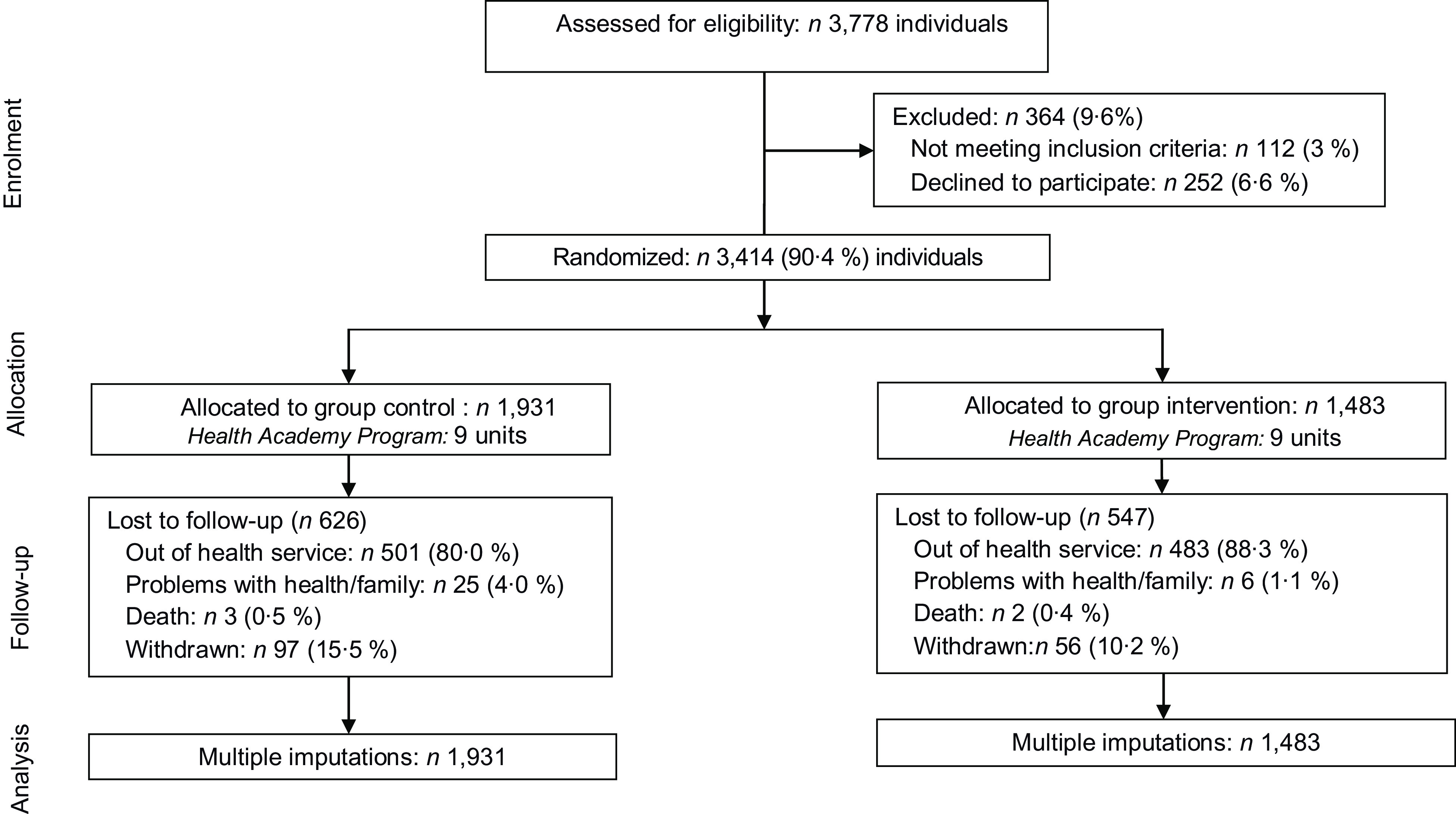
Flowchart of the study design

Of the HAP centres sampled, 3778 users (age, ≥ 20 years) participated in routine service activities (such as physical exercise programmes) in the preceding month. Pregnant women and individuals with cognitive difficulties (preventing attentive research participation) were excluded from the study. Considering 6·6 % (*n* 252) exclusions and 3·0 % (*n* 112) refusals, 3414 individuals participated in this study, with a corresponding study response rate of 90·4 %^(19)^. Figure 1 shows a flowchart of the study design.

### Nutritional intervention

Individuals in the IG and CG took part in routine activities of the provided service, which included 1-h physical activity sessions, 3 times a week. Additionally, a nutritional intervention was performed with the IG to promote FV intake during the 7-month period. The participants in the CG did not receive any intervention specific to FV intake during the same period^(20)^.

**Fig. 2:**
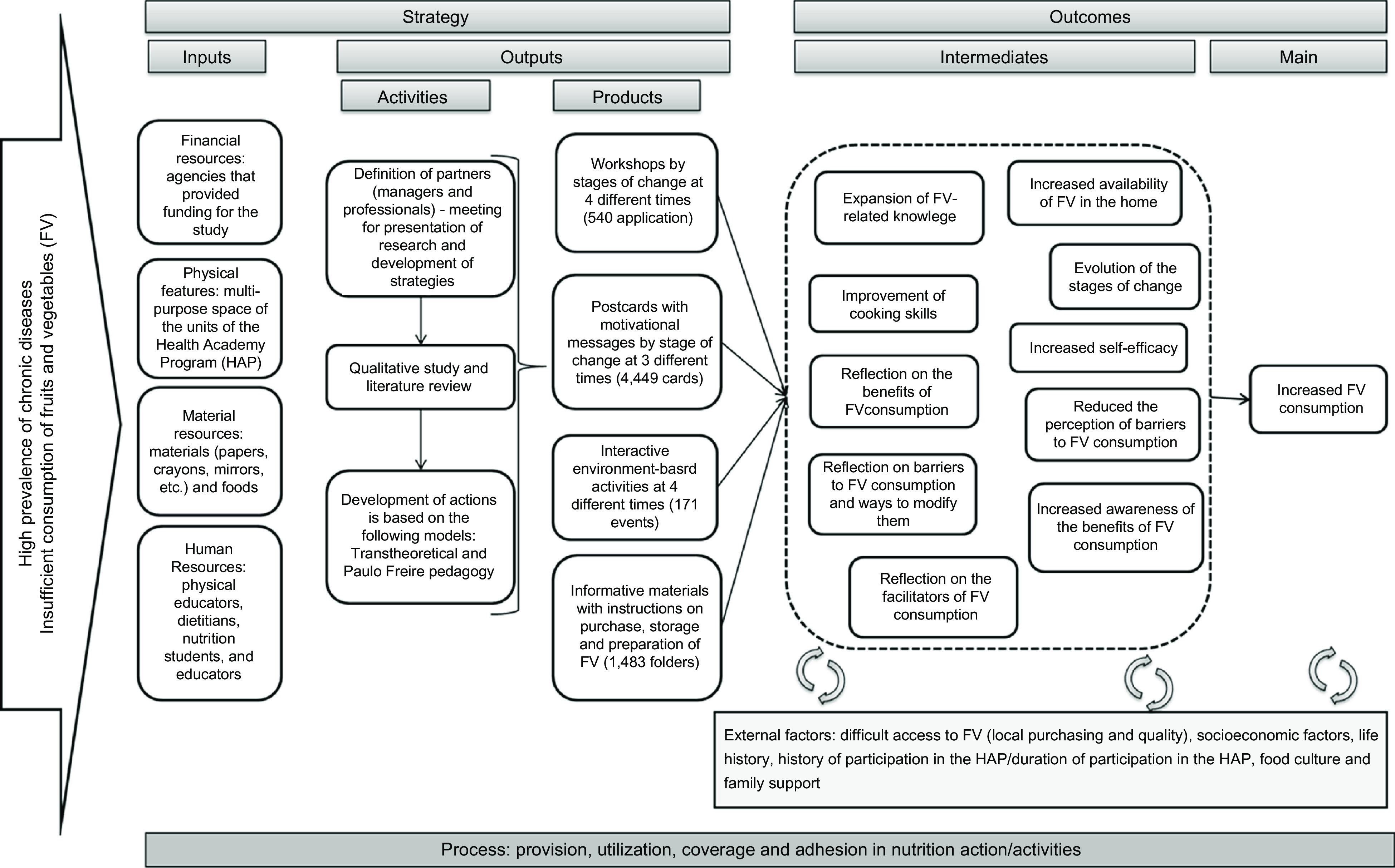
Logic model for the development of nutritional intervention

The intervention for promoting FV intake was developed by an interdisciplinary team comprising nutritionists, educators and psychologists. In addition, a trained team of 3 nutritionists performed the educational activities^(20)^. We used a logic model to plan and evaluate the nutritional intervention (Fig. 2).

The intervention was based on the transtheoretical model^(14)^ and Paulo Freire’s problematising dialogic pedagogy^(21)^. This model facilitated the planning and implementation of actions according to individual characteristics including perception, readiness, attitude and motivation to undergo behavioural changes^(14)^. The model included 4 pillars: (1) stages of change; (2) processes of change; (3) self-efficacy and (4) decisional balance. The stages of change defined phases that involved action planning and development, and included, pre-contemplation: individuals did not intend on changing their behaviour in the foreseeable future; contemplation: individuals began to identify the need for change; preparation: individuals were ready to change their behaviour within 30 d; action: individuals were capable of short, immediate changes for a period of up to 6 months; maintenance: an individuals’ behaviour changed > 6 months prior, requiring the prevention of relapse and consolidation of gains^(14)^.

The cognitive and behavioural processes of change underlined the understanding of how change occurs during the different stages. The educational activities focused on increasing awareness of behaviour and its consequences. Interventions were also aimed to increase an individuals’ confidence in their ability to achieve the desired behaviour when faced with obstacles (self-efficacy) and to increase awareness of the benefits of a healthy diet while minimising the factors against change (decisional balance)^(14)^.

Problematising dialogic pedagogy, proposed by Paulo Freire, was chosen as a theory to guide the intervention development, to advance the transtheoretical model and advocate autonomy and empowerment. In this theory, education creates the possibility of liberation, which promotes conscientious attitudes and presumes a horizontal and dialogical relationship between participants, which is aimed at knowledge exchange instead of a relationship in which one is superior to the other^(21)^.

To define the subject matters on FV to be studied, a qualitative pilot study was conducted to examine social representations associated with FV intake^(22)^. This study identified the priority categories including food portions; nutritional information on FV and obstacles including cost, flavour, reduced family support, the time required to buy and prepare food and difficulties in shopping for good quality FV.

Four educational strategies were used: (1) group educational sessions, with up to 20 participants each; (2) motivational cards; (3) environment-based activities (e.g. movies and cooking competitions) and (4) informational materials. The cards and informational materials were delivered at the HAP centres with the assistance of healthcare professionals^(20)^.

The educational sessions and motivational cards varied across different stages of change with respect to FV intake. Each group was divided according to stages of change^(20)^. Thus, the participants were regrouped into: (1) pre-action (pre-contemplation and contemplation stages: individuals are not ready to change although they required action to shape their motivation); (2) decision (determination stage: individuals were ready to change their behaviour within 30 d) and (3) action (action and maintenance stages: individuals were capable of short, immediate changes, although the prevention of relapse and consolidation of gains was required)^(14,20)^.

The intervention consisted of 4 group educational sessions monthly, 3 cards for each stage of change, 3 environment-based activities and 1 informational material; a total of 11 actions. To monitor attendance, we recorded the names of all participants who took part in each of the strategies. We sent workshop invitations to the participants by post and invited them through phone calls^(20)^.

### Outcome assessment: FV intake

The outcome measure in this study was the change in FV intake (grams) during a follow-up of 7 months after the intervention (FV intake at 7 months – FV intake at baseline).

FV intake was assessed using questions (a brief questionnaire of FV intake evaluation) adapted from the National Risk Factor Surveillance System for Chronic Diseases^(23)^ and validated in a previous study (correlation coefficients for the intake of fruits, vegetables and both FV were 0·44, 0·34, 0·37, respectively; when compared with the 24-h recall)^(24)^.

The participants were asked how often they consumed fruits (excluding juice) and vegetables (excluding potatoes and cassavas), their portion sizes (units for fruits and the numbers of tablespoons for vegetables) and preparation mode (for vegetables). The frequency of FV intake was reported in 6 categories: (1) never; (2) almost never; (3) 1–2 d/week; (4) 3–4 d/week; (5) 5–6 d/week or (6) every day^(19,24)^. For this, the QBrief-FV comprised 7 direct questions regarding the frequency of FV consumption. For fruit consumption, it was asked: ‘How many days a week do you usually eat fruit?’, followed by a question regarding the amount consumed (units, slices and size): ‘On an ordinary day, how many servings of fruits do you eat’ and ‘On how many days of the week, do you usually eat at least one type of vegetable?’ Next a question regarding quantities (tablespoons): ‘On an ordinary day, how many spoons (soup) of vegetables do you eat?’, in addition to the mode of consumption (raw or cooked)^(19,24)^.

Daily FV intake was estimated by multiplying the portion size by intake frequency (d/week) divided by 7 d (week). FV intake was calculated as the sum of the daily FV portions. FV intake (expressed in grams) was calculated by multiplying the portion of FV by 80 g^(25,26)^:

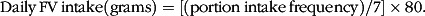




The analyses were performed according to total FV intake (g/d) and separately for fruit (g/d) and vegetable (g/d) intake, considering that WHO recommends 400 g of FV daily^(1)^, and the intake of nearly 200 g of each food group can be considered adequate, for example, 240 g of vegetables and 160 g of fruits daily^(25,26)^. Furthermore, it is possible that the population has different perceptions and behaviours related to FV intake^(22)^.

### Secondary outcomes

The secondary outcome measures included behavioural variables (i.e. stages of change, self-efficacy and decisional balance) and knowledge of FV crops (yes or no)^(19)^.

The stages of change were measured using an algorithm for the intake fruits and vegetables, separately. This algorithm assessed the readiness to change intake with the following response options: (1) pre-contemplation (not intending to increase FV intake in the next 6 months); (2) contemplation (intending to increase FV intake in the next 6 months); (3) preparation (intending to increase FV intake in the next 30 d); (4) action (current adequate FV intake for <6 months) and (5) maintenance (current adequate FV intake for at least 6 months). For analysis, the participants were regrouped into 3 groups: pre-action, preparation and action^(14)^.

Self-efficacy was used to assess a participant’s confidence to change FV intake. Participants rated their confidence in 4 situations on a 5-point Likert scale (1 = not at all confident to 5 = extremely confident). The 4 situations were: ‘fruits and vegetables are easy to buy in my neighbourhood’; ‘I can buy a variety of fruits and vegetables even when they are expensive’; ‘I can consume the amount recommended of fruits and vegetables’; ‘I have time to prepare/consume fruits and vegetables, even on days when I am in a hurry’^(27)^. These were summed, with a higher score indicating a greater confidence.

The decisional balance measured a participant’s perception of the benefits and barriers to FV intake. The question had 4 items regarding the benefits and 4 items regarding the barriers to FV intake^(28)^. Participants rated the importance of each item on a 5-point Likert scale (1 = completely disagree to 5 = completely agree). Responses were summed separately for each category, with a higher score indicating a higher perceived benefit or barrier^(17)^.

For knowledge on FV crops, participants were asked: ‘Do you know what a FV crops is?’ We include this data in the analysis, as one of the main obstacles, reported by a qualitative study, in increasing the consumption of FV, was the high cost. In Brazil, when food is at the crop, they are generally cheaper, which may favour higher consumption of FV, especially in vulnerable populations. Additionally, increasing self-efficacy, improving a participant’s knowledge and ability to make plans and changing perceptions (benefits and barriers) could also aid in increasing the intake of FV.

### Covariables

We investigated the following demographic variables: sex, age, marital status (married, single, divorced and widowed), educational level (0–8 years or >8 years), economic classes based on the Brazilian economic classification criteria (D/E-worst, C and A/B-best), occupation status (housewife, retired, unemployed and employed) and time of participation in HAP (years). The Brazil Economic Classification Criterion estimates the purchasing power of urban people and families. The categorisation of a professional occupation was performed according to the profile of the program’s users^(19)^. The demographic profiles were analysed based on Brazilian national studies^(6)^.

Additionally, we used anthropometric measurements (weight and BMI)^(19)^. Weight was measured with a calibrated digital scale, with an accuracy of 0·1 kg. Height was measured using a stadiometer with a fixed vertical backboard and an adjustable headboard, with an accuracy of 0·1 cm. The weight and height were measured with individuals wearing light clothing and barefoot, without props or objects on their head or in their pockets.

All data were collected in a face-to-face manner by nutrition students and nutritionists who were periodically trained by the researchers. A pilot study identified the adequacy of the research instruments for the population^(19)^.

### Statistical analyses

All analyses were performed using Stata/se, Version 13.1 (StataCorp., LP) and R software version 20. All *P*-values were 2-tailed and considered statistically significant if < 0·05.

Incomplete data (*n* 815) on FV intake were imputed using multiple imputations by chained equations to avoid a loss of power. Differences in results with and without multiple imputations for those participants were minimal and non-significant. For the multiple imputation methods used in this study, it was assumed that the missing data occurred randomly (missing at random – MAR), that is, that the losses were not correlated with any variable and were not due to a selection bias. In this case, the estimates for the missing were obtained using only the observed data. The Multivariate Imputation by Chained Equations (MICE) package, from software R, was used.

To evaluate the effectiveness of the 7-month intervention, we analysed the main outcome, change in FV intake, separately and together, after 7 months of intervention (12 months from baseline), and secondary outcomes that included knowledge of FV crops, stages of change, self-efficacy and the decisional balance for FV intake.

Characteristics at baseline were presented as means and SDs. The differences between groups (CG *v.* IG) at baseline were assessed using *χ* ^2^ and Student’s *t*-tests for independent samples. The secondary outcomes were assessed at baseline and after 7 months (between-group differences) using *χ* ^2^ and Student’s *t*-tests for independent samples and within-group differences after 7 months (baseline *v.* follow-up) were determined using McNemar’s and paired Student’s tests.

The change in FV intake was treated as a continuous variable. The sample was divided into quartiles according to total FV, quartiles of vegetable and fruit intake (g/d) at baseline; the fourth quartile included the highest consumption.

A generalised estimating equation was used to examine the main outcome: FV and separately for fruits and vegetables intake. This analytic method was used to examine the associations of time-varying and time-constant independent variables with a time-varying dependent variable (e.g. FV intake). Assessments were conducted before the intervention and 7 months after (follow-up). Results were adjusted for baseline variables that were distributed differently among study participants and lost to follow-up, such as sex, age, education, time of participation in HAP, income/economic classes (based on the Brazilian economic classification criteria) and BMI.

## Results

Of the 3414 participants included at baseline, 65·6 % (*n* 2241) completed the trial: 1305 in the CG and 936 in the IG (Fig. 1). Losses to follow-up were more common among women, younger people, those with a higher education and those in the action stage. However, there were no differences in FV intake between retained participants and those lost to follow-up (372·9 g/d *v*. 367·6 g/d, *P* = 0·21).

The participants had a mean age of 57·2 years (95 % CI 56·7, 57·5) and the majority was women (88·1 %) and had taken part in the HAP for approximately 2 years. The intervention and CGs had similar baseline characteristics, except for age and time of participation in the HAP (Table 1).

**Table 1:** Baseline characteristics of study participants

Characteristics	Control group (*n* 1931) %	Intervention group (*n* 1483) %	*P*-value
Sex			0·07*
Female	88·9	86·9	
Male	11·1	13·1	
Age (years)			
Mean	57·6	56·6	0·02†
95 % CI	57·1, 58·1	56·0, 57·3	
Marital status			0·71*
Married	61·9	61·1	
Divorced	7·8	8·9	
Single	14·1	14·2	
Widowed	16·1	15·8	
Education			0·12*
0–8 years	55·5	58·2	
>8 years	44·5	41·8	
Economic classes‡			0·71*
A/B ($694·20/family/month)	29·5	29·5	
C ($360·10/family/month)	54·6	55·6	
D/E ($218·47/family/month)	15·9	14·9	
Occupation			0·13*
Housewife	28·6	28·7	
Retired	36·8	36·6	
Unemployed	1·5	2·6	
Employed	33·1	32·0	
Time of participation in HAP (years)			
Mean	1·82	1·46	< 0·01†
95 % CI	1·74, 1·91	1·39, 1·52	
Anthropometrics			
Weight (kg)			
Mean	68·9	69·4	0·31†
95 % CI	68·3, 69·5	68·7, 70·1	
BMI (kg/m^2^)			
Mean	27·8	27·9	0·73†
95 % CI	27·6, 28·1	27·7, 28·1	
Stage of change groups			0·13*
Pre-action	16·2	18·8	
Decision	36·0	34·7	
Action	47·8	46·5	
Fruits (g/d)			
Mean	153·7	144·6	0·06†
95 % CI	148·3, 159·1	138·1, 151·0	
Vegetables (g/d)			
Mean	227·8	232·8	0·19†
95 % CI	223·0, 232·6	226·8, 238·8	
FV consumption (g/d)			
Mean	373·1	366·9	0·33†
95 % CI	365·1, 381·1	357·1, 376·6	
Quartiles of FV consumption (g/d)			0·22*
Q1			
Mean	167·2	162·0	
95 % CI	161·8, 172·6	156·3, 167·8	
Q2			
Mean	309·9	308·9	
95 % CI	306·9, 313·0	305·3, 312·5	
Q3			
Mean	429·7	425·2	
95 % CI	426·7, 432·7	421·5, 428·9	
Q4			
Mean	633·9	644·3	
95 % CI	619·6, 648·3	627·1, 661·4	

FV, fruit and vegetable; HAP, health academy programme; Q, quartile.

* Chi-squared test.

† Student’s *t*-test.

‡ Based on the Brazilian Economic Classification Criteria (estimate of family income per month).

Data are presented as percentages or means (95 % CIs).

At baseline, the average daily FV intake was 370·4 g/d (95 % CI 364·2, 376·6), fruits were 149·7 g/d (95 % CI 145·6, 153·9) and vegetables 229·9 g/d (95 % CI 226·8, 238·8) (Table 1). The participants who consumed < 80 g/d (≅ 1 portion), < 160 g/d (≅ 2 portions), and ≥400 g/d of FV (≥ 5 portions) accounted for 3·1 %, 10·3 % and 42·9 %, respectively. The main fruits consumed were bananas, oranges and apples, and vegetables were tomatoes, lettuce and carrots^(29)^.

There was a loss of follow-up of 36·9 % in the IG, and the main reasons reported for non-attendance included disengagement from the health service (60·5 %), employment or studying (13·6 %) and health or family problems (8·0 %). This study intervention offered 11 different actions to the IG, and approximately 90 % of the participants took part in at least 5 types of educational activities resulting in a 60 % adherence.

Regarding the secondary outcomes, participants in the IG showed progression in the stages of change, the percentage of those in the pre-action and decision stages decreased, whereas the percentage of those in the action stage increased (*P* < 0·001). In addition, we identified increased self-efficacy levels (*P* < 0·001) and improved knowledge of FV crops, and none of these changes were identified in the CG (Table 2).

**Table 2:** Baseline values and changes after 7 months of follow-up within and between the control and intervention groups according to secondary outcomes including stages of change, self-efficacy and decisional balance

Variables	Control group							Intervention group								
	Baseline		Follow-up		Change		*P*-value	Baseline		Follow-up		Change			Between-group: baseline	Between-group: after 7 months of follow-up
	Mean	95 % CI	Mean	95 % CI	Mean	95 % CI		Mean	95 % CI	Mean	95 % CI	Mean	95 % CI	*P*-value	*P*-value	*P*-value
Stage of change groups (%)							0·46†							<0·001†	0·13§	<0·001§
Pre-action	16·2		15·4		−0·7	−2·7, 1·2		18·8		12·4		−6·4	−8·6, −4·2			
Decision	36·0		27·5		−8·5	−11·0, −6·1		34·7		18·5		−16·2	−18·9, −13·4			
Action	47·8		57·1		9·3	6·8, 11·7		46·5		69·1		22·6	19·8, 25·3			
Self-efficacy*	8·9	8·7, 9·1	8·8	8·7, 9·0	−0·02	−0·2, 0·1	0·62‡	8·6	8·5, 8·8	9·1	8·9, 9·3	0·5	0·3, 0·7	<0·001‡	0·03‖	0·01‖
Decisional balance																
Benefits	12·6	12·5, 12·7	12·6	12·5, 12·7	0·01	−0·1, 0·1	0·76‡	12·4	12·3,12·5	12·5	12·3, 12·6	0·05	−0·1, 0·2	0·52‡	0·01‖	0·03‖
Barriers	6·1	5·9, 6·2	6·1	5·9, 6·2	−0·0	−0·2, 0·1	0·97‡	6·2	6·1, 6·3	6·0	5·9, 6·2	−0·2	−0·3, 0·0	0·06‡	0·41	0·37‖
FV crops (%)	78·2		76·4		−1·7	−3·9, 0·4	0·12†	70·9		77·2		6·3	3·6, 9·1	<0·001†	<0·001§	0·59§

* Self-efficacy and decisional balance items were scored on a 5-point scale from 1 to 5 (maximum score of 20). For benefits, higher scores indicate greater perceived benefits; lower scores indicate smaller perceived barriers.

† McNemar test.

‡ Paired Student test.

§ Chi-squared test.

‖ Student’s *t*-test.

Data are presented as percentages or means (95 % CIs).

The multivariate analysis of the evolution of FV intake, after 7 months of nutritional intervention (the main outcome), revealed that in the lowest quartile of FV intake, participants in the IG consumed more FV than those in the CG (+23·4 g/d; (95 % CI 6·7, 40·0); *P* = 0·01). However, those who had the highest consumption, and the highest quartile of FV at baseline, reduced their intake after the intervention, when the IG was compared with the CG (−32·1 g/d; (95 % CI −60·1, −4·1); *P* = 0·03). However, the consumption was within the recommended daily amount of 400 g. When we analysed the consumption of fruits and vegetables separately, we observed a reduction in the consumption of vegetables in the total sample and among those with high consumption (fourth quartile), but no change in the other quartiles of vegetable intake. For fruits, participants had the lower intake of fruits at baseline increased their intake after intervention (+17·3 g/d; (95 % CI 5·1, 29·4); *P* = 0·01), when the IG was compared with the CG (Table 3).

**Table 3:** Change in daily fruit and vegetable consumption according to an intervention

FV consumption (g/d)	Control						Intervention						*P*-value				
	Baseline (g/d)		Follow-up (g/d)		Mean differences		Baseline (g/d)		Follow-up (g/d)		Mean differences			Between-group difference*		Between-group difference*,†	
	Mean	95 % CI	Mean	95 % CI	Mean	95 % CI	Mean	95 % CI	Mean	95 % CI	Mean	95 % CI		Mean	95 % CI	Mean	95 % CI
Fruits	153·7	148·3, 159·1	158·4	153·2, 163·6	4·7	−0·8, 10·2	144·6	138·1, 151·0	163·1	157·1, 169·0	18·5	11·5, 25·5	13·8	4·9, 22·7	13·8	4·9, 22·7	0·002‡
Q1	11·9	10·5, 13·3	86·3	78·1, 84·5	74·4	66·3, 82·5	12·0	10·5, 13·5	103·1	93·8, 112·5	91·1	81·8, 100·4	16·6	4·3, 28·9	16·7	4·4, 29·0	0·01‡
Q2	115·6	112·7, 118·5	143·1	135·4, 150·8	27·5	19·8, 35·2	115·3	112·0, 118·6	160·0	150·8, 169·3	44·8	35·3, 54·2	17·2	5·1, 29·5	17·3	5·1, 29·4	0·01‡
Q3	232·8	231·3, 234·3	207·5	198·0, 217·0	−25·3	−34·7, −15·9	233·6	231·9, 235·4	208·9	197·7, 220·0	−24·8	−35·9, −13·6	0·6	−14·0, 15·1	0·6	−14·0, 15·1	0·94
Q4	371·0	358·0, 383·9	237·0	222·1, 251·9	−134·0	−151·3, −116·6	378·4	358·4, 398·4	224·2	204·7, 243·7	−154·2	−181·1, −127·3	−20·2	−52·0, 11·5	−20·2	−52·0, 11·5	0·21
Vegetables	227·8	223·0, 232·6	212·7	208·4, 217·1	−15·1	−20·6, −9·6	232·8	226·8, 238·8	207·3	202·2, 212·4	−25·5	−32·2, −18·8	−10·5	−19·1, −1·8	−10·4	−19·1, −1·8	0·02‡
Q1	132·9	130·6, 135·3	182·4	176·5, 188·2	49·4	43,4, 55·3	131·7	128·9, 134·4	179·3	171·8, 186·8	47·6	40·1, 55·1	−1·8	−11·3, 7·8	−1·8	−11·3, 7·8	0·72
Q2	193·4	192·4, 194·4	205·1	195·5, 214·7	11·6	2·1, 21·2	192·5	191·3, 193·7	202·7	191·4, 214·0	10·2	−1·21, 21·6	−1·4	−16·2, 13·4	−1·4	−16·3, 13·4	0·85
Q3	247·2	245·6, 248·9	225·2	217·2, 233·1	−22·1	−30·0, −14·1	248·1	246·2, 250·1	217·0	206·9, 227·1	−31·1	−41·2, −20·9	−9·0	−21·9, 3·8	−9·0	−21·9, 3·8	0·19
Q4	393·4	382·8, 403·9	253·8	241·8, 265·8	−139·5	−154·6, −124·4	404·6	392·0, 417·1	243·3	231·4, 255·2	−161·3	−176·4, −146·1	−21·7	−43·0, −0·4	−21·7	−43·0, −0·4	0·05‡
Total FV	373·1	365·1, 381·1	362·2	333·3, 365·1	−10·9	−18·3, −3·5	366·9	357·1, 376·6	361·7	354·1, 369·3	−5·2	−14·4, 4·0	5·7	−6·1, 17·5	5·6	−6·2, 17·5	0·35
Q1	167·2	161·8, 172·6	255·9	244·9, 266·8	88·7	77·6, 99·7	162·0	156·2, 167·8	274·1	261·6, 286·5	112·1	99·6, 167·8	23·4	6·7, 40·0	23·4	6·7, 40·0	0·01‡
Q2	309·9	306·9, 313·0	336·0	324·9, 347·1	26·1	15·1, 37·1	308·9	305·3, 312·5	346·9	333·6, 360·2	37·9	24·5, 51·4	11·8	−5·9, 29·2	11·8	−5·4, 29·2	0·18
Q3	429·7	426·7, 432·7	400·2	388·9, 411·5	−29·5	−40·7, −18·3	425·2	421·5, 428·9	398·6	385·2, 411·9	−26·7	−39·9, −13·4	2·8	−14·5, 20·1	2·8	−14·5, 20·1	0·75
Q4	633·9	619·6, 648·3	476·0	460·3, 491·7	−157·9	−176·9, −138·9	644·3	627·1, 661·4	454·4	437·9, 470·8	−189·9	−210·7, −169·1	−31·9	−60·0, −3·9	−32·1	−60·1, −4·1	0·03‡

FV, fruit and vegetables; Q, quartile.

* Coefficient (*β*) (95 % CI).

† Adjusted for sex, age, education (years), attending the programme (years), Brazilian economic classification and BMI.

‡ Statistically significant > 0 05.

Data are presented as means (95 % CIs).

## Discussion

The nutritional intervention was effective in increasing total FV intake and fruits intake among individuals with a lower intake at baseline and in maintaining intake among those who reported consuming FV as recommended (400 g/d), mainly fruits. Additionally, participants in the IG showed improvements in the stage of change (action stage increased), self-efficacy, perceived benefits of FV intake and knowledge of FV crops, which may produce additional changes in FV intake.

The participants with lowered FV intakes in the IG had an average increase of 112 g (1·4 servings), which was higher than that reported in other studies. These results show that nutritional interventions can contribute to changes in FV intake. According to a systematic review of interventions, based on feeding behaviour theories, FV intake increased by 1·13 servings (≅90 g/d) among healthy adults, compared to only 0·97 servings (≅78 g) among adults with a low-income and those from minority groups^(12)^. A meta-analysis on behavioural interventions demonstrated that FV intake increased by 68·6 g/d after 3 months of intervention and by 65·8 g/d for interventions >3 months^(30)^. Another systematic review, on the effects of nutrition care provided by primary health professionals to adult patients, showed significant improvements in dietary behaviours, such as increased daily intake of foods including FV, high-fibre bread and fish. However, certain studies did not find any improvement in FV intake, and one study indicated a reduction in daily FV intake^(31)^.

Individuals appear to increase their FV intake depending on their intake at the baseline. People with a low intake increased their FV intake (quartile 1), adequate intake was maintained (quartiles 2 and 3) and those with high intake reduced their FV intake (quartile 4), although they still met the recommendation of 400 g/d^(1,26)^. When analysed separately, we observed an increase in fruit consumption, especially among those with low consumption (quartiles 1 and 2) and a reduction in vegetable consumption among those with higher consumption (quartile 4), following the same trend when analysing the consumption of fruits and vegetables together. These results are consistent with WHO recommendations, and it appears that the intervention achieved the purpose of increasing knowledge, empowering participants and promoting conscientious attitudes^(21)^. The participants were able to identify a need for change (intake lower or higher than the recommendation), especially those with an intake below the recommendation.

An increase of one FV serving (80 g/d) can reduce the risk of all-cause mortality by 5 % and the risk of CVD mortality by 4 %^(2)^; the intake of even 3 servings per day (375 g/d) has health benefits^(32)^. A systematic review of meta-analyses showed that protective associations significantly increased the intakes of 200 g/d but slightly increased or decreased the intakes of >300 g/d^(33)^.

It is necessary to emphasise that FV intake was high at baseline in both the IG and CG. Two-fifths of the participants in the IG reached the recommended daily intake of 400 g, and the average fruit intake was higher than the Brazilian and international data^(4–6)^. A study that included 143 305 adults from 18 countries in 5 continents across income levels showed that the mean FV intake was 2·14 servings (≅171 g) in low-income countries^(7)^. In addition to high fruit consumption, the intake in the CG also increased in our study compared with Brazilian data (baseline)^(23)^. A possible explanation is that participating in a health service can promote healthy eating, including FV intake, and the nutritional intervention enhances the adoption of healthy eating. This results in an adequate FV intake among the majority of HAP participants living in poor communities and raises the hypothesis that the primary care service model used in HAP may be a way to overcome the global challenge of increasing FV intake.

In addition, improvements in self-efficacy, knowledge of FV crops, stages of change and perceived benefits of FV intake may support additional changes in FV intake among the participants over a long-term period. This study presents the methodology of an innovative intervention for the promotion of FV intake among adults, based on an individuals’ perceptions of their consumption habits. Evidence suggests that stages of change, self-efficacy, social support and knowledge are predictors of FV intake^(14,34,35)^. Studies have shown that individuals in action and maintenance stages are more likely to follow diets than individuals in the pre-contemplation, contemplation and preparation stages; moreover, their FV intake is likely to be higher^(14,35,36)^. The increase in self-efficacy and perceived benefits can also be reflected in the progression of the stages of change and the increased number of people in the action group^(36)^. Nutritional interventions should include education on the health benefits of healthy food and actions that increase self-efficacy, improve a participant’s ability to make plans and change behaviours to increase FV intake^(35)^.

FV intake appears to be higher among individuals with a higher self-efficacy and a better perception of the food environment^(37)^. The consumer’s food environment, in turn, could affect FV intake as the access to commercial establishments appeared to be important for the quantitative intake^(29)^. To reach the recommended intake, as verified here, we believe that the educational activities should be developed and sustained based on scientific evidence. Furthermore, these activities should cover information regarding improved accessibility, availability and quality of FV.

This intervention achieved positive outcomes because of the planning of inputs, the logic model for the development of the nutritional intervention, activities and expected results, as well as the involvement of healthcare professionals^(38,39)^. Additionally, the primary care services are strategic for the development of nutritional interventions because they can provide additional social support among individuals by improving their knowledge. Therefore, a nutritional intervention can contribute to increasing self-efficacy and reducing perceived barriers according to the intention and readiness to change food intake habits.

One issue that may have prevented us from achieving better results with the intervention was the high percentage (42·9 %) of participants reporting adequate FV intake at baseline. This may be partly because the study was conducted with participants in the HAP, a programme that aims to promote healthy lifestyles. However, previous studies on these services showed that service users with inadequate FV intake accounted for 60–75 %^(19,29)^; these low percentages were among the motivations for this work. But, these studies only evaluated participants when they started participating in the HAP; and our study covered all users, regardless of the time of participation in the HAP, which possibly helped to show how much the HAP can contribute to healthy eating. In the general population, FV intake may be lower, and the change after an intervention could be greater. Given the demand for additional and improved information on the effectiveness of actions under real-life conditions and the potential to contribute to greater sustainability of results, this study highlights the need to conduct further studies, mainly in the health services.

The results may have been affected by the differences between the 2 groups at baseline and by follow-up losses. The adjustments in the analysis and multiple imputations were used to minimise these possible problems. Segmental losses occurred mainly among women, young people and people with a higher education. However, all analyses were adjusted accordingly to minimise the impact on the results. This was likely because of the opening hours of service in the morning, which may have hindered the adherence among economically active individuals^(19,20,39)^, but the adherence was higher than national and international values namely, 22·7 % to 56·0 %^(36)^. In addition, we implemented all the possible ways to ensure a high retention rate of our participants such as maintaining contact through phone calls. In addition, we evaluated differences in baseline characteristics of those participants lost during follow-up versus those retained in the study. Differences in FV intake between lost and retained participants were minimal and NS (CG *P*-value = 0·45 and IG *P*-value = 0·32). To reduce the uncertainty of variation between pre- and post-intervention, we adjusted for possible confounding factors (sex, age, education, participation in the HAP, and BMI); and longitudinal analysis was performed to determine the effects of the intervention on controlling for similarities among individuals and FV intake.

The difficulty in obtaining reliable measures of food intake has been identified as a limiting factor in the literature. Inherent to our methodology, there is the potential for some degree of misclassification of FV intake. It cannot be excluded that self-reports of FV intake are susceptible to substantial social approval bias. As previously indicated, a greater subject-specific bias existed in self-reported FV intake in the CG compared to the treatment group^(40)^. In summary, self-reported information could have led to some degree of overestimation or underestimation of the effectiveness of the intervention. However, QBrief-FV has been previously validated, and we used the same questionnaire at baseline and follow-up that was provided by trained staff, and professionals involved in the intervention did not participate in the data collection at the intervention Primary Health Care services. We emphasise that all data were collected by nutrition students and nutritionists trained periodically. Data collection procedures were standardised, and protocols were used to minimisse errors and biases. This contributes to the increased reliability of our data.

Caution is required regarding the external validity of the data. HAP participants are regular users of the service aimed at health promotion, with a focus on physical activity; therefore, they may be more health conscious and have a higher average FV intake than the general population. Caution is required when extrapolating our results to the general population because the participants attend a service aimed at promoting health and focusing on the practice of physical exercise, which renders the participants more aware of their health. However, we believed that the results from this study could be extrapolated to other populations of Primary Health Care users. We, therefore suggest that the intervention be tested in other population groups in order to verify its effectiveness.

It should be noted that all actions (group educational sessions, motivational cards, environment-based activities and informational materials) were designed to be reapplied in primary care and all materials prepared by the research was made available for primary care with instructions on how to apply. Other strengths of this study include the design, the large sample size and the use of validated methods to assess consumption of FV, all data were collected in a face-to-face manner by nutrition students and nutritionists who were periodically trained. A pilot test identified the adequacy of the research instruments for the population studied. Adherence to the intervention was similar or higher than other studies, and the methodology of the intervention appeared to contribute to adherence^(39)^. The intervention was effective among people who had a low FV consumption, mainly fruits, but the consumption, within the recommended guideline (400 g/d), was maintained among the other participants. Thus, we hypothesise that the nutritional intervention should be carried out according to the demands and needs of the population.

In conclusion, this was a randomised trial for FV promotion held by the health service system with a low-income population. Our results suggest that the nutritional intervention, based on the perception of intake and group activities, was effective in increasing FV intake among individuals with low total FV intake, mainly fruits, and maintaining FV intake among those with sufficient FV intake. The increase in knowledge, self-efficacy and perception of benefits corroborates that a nutritional intervention could be considered a useful tool in promoting FV intake. However, to confirm the study’s results, we suggest that future studies should be conducted over a longer period in different contexts, with a focus on analysing the maintenance of these changes.
